# Temperature Modulates Coccolithophorid Sensitivity of Growth, Photosynthesis and Calcification to Increasing Seawater *p*CO_2_

**DOI:** 10.1371/journal.pone.0088308

**Published:** 2014-02-05

**Authors:** Scarlett Sett, Lennart T. Bach, Kai G. Schulz, Signe Koch-Klavsen, Mario Lebrato, Ulf Riebesell

**Affiliations:** 1 Biological Oceanography, GEOMAR Helmholtz Centre for Ocean Research Kiel, Kiel, Germany; 2 Centre for Coastal Biogeochemistry, School of Environmental Science and Management, Southern Cross University, Lismore, Australia; University of Hong Kong, Hong Kong

## Abstract

Increasing atmospheric CO_2_ concentrations are expected to impact pelagic ecosystem functioning in the near future by driving ocean warming and acidification. While numerous studies have investigated impacts of rising temperature and seawater acidification on planktonic organisms separately, little is presently known on their combined effects. To test for possible synergistic effects we exposed two coccolithophore species, *Emiliania huxleyi* and *Gephyrocapsa oceanica*, to a CO_2_ gradient ranging from ∼0.5–250 µmol kg^−1^ (i.e. ∼20–6000 µatm *p*CO_2_) at three different temperatures (i.e. 10, 15, 20°C for *E. huxleyi* and 15, 20, 25°C for *G. oceanica*). Both species showed CO_2_-dependent optimum-curve responses for growth, photosynthesis and calcification rates at all temperatures. Increased temperature generally enhanced growth and production rates and modified sensitivities of metabolic processes to increasing CO_2_. CO_2_ optimum concentrations for growth, calcification, and organic carbon fixation rates were only marginally influenced from low to intermediate temperatures. However, there was a clear optimum shift towards higher CO_2_ concentrations from intermediate to high temperatures in both species. Our results demonstrate that the CO_2_ concentration where optimum growth, calcification and carbon fixation rates occur is modulated by temperature. Thus, the response of a coccolithophore strain to ocean acidification at a given temperature can be negative, neutral or positive depending on that strain's temperature optimum. This emphasizes that the cellular responses of coccolithophores to ocean acidification can only be judged accurately when interpreted in the proper eco-physiological context of a given strain or species. Addressing the synergistic effects of changing carbonate chemistry and temperature is an essential step when assessing the success of coccolithophores in the future ocean.

## Introduction

Rapidly increasing fossil fuel emissions and deforestation over the past 250 years have increased atmospheric CO_2_ concentrations at an unprecedented pace and caused a rise in global average temperatures as well as changes in ocean carbonate chemistry [1], [2]. Presently, the ocean takes up more than 1/4 of the anthropogenic CO_2_ emissions, thereby mitigating global warming but changing the ocean's carbonate chemistry equilibrium towards a decrease in carbonate ions [CO_3_^2−^] and pH (i.e. ocean acidification, OA) [2], [3] and an increase in bicarbonate ions [HCO_3_^−^] and [CO_2_] (i.e. ocean carbonation). Despite the massive sequestration of anthropogenic CO_2_ in the oceans, global warming will still most likely result in an average temperature increase between 1 and 6°C by the end of this century [1]. Ocean warming is expected to influence metabolic rates of marine organisms with unforeseen consequences for marine biogeochemical cycling and ecosystem functioning [4] [5].

Coccolithophores are single-celled autotrophic phytoplankton capable to precipitate calcium carbonate (CaCO_3_) as small scales (coccoliths) to cover the organic part of the cell. They are considered the most important calcareous primary producers contributing ∼1–10% to today's oceanic primary production [6] and ∼50% to CaCO_3_ found in pelagic sediments [7]. Hence, they are key players in marine biogeochemical cycles [8], [9]. The most important bloom-forming coccolithophore species are *Emiliania huxleyi* [10], [11], [12] and *Gephyrocapsa oceanica* [13]. Both species have the ability to produce extensive blooms which are detectable from space [14]–[18]. In modern oceans, *E. huxleyi* is the most abundant species [19], but its contribution to CaCO_3_ sedimentation is relatively small owing to its low coccolith weight [20]. *G. oceanica* is generally less abundant than *E. huxleyi* but its contribution to CaCO_3_ export into the deep is larger because its coccoliths contain significantly more CaCO_3_ [21].

During the last 15 years, coccolithophores have gained considerable attention due to the sensitivity of calcification to changing seawater *p*CO_2_ [22]. To date, experiments investigating the effects of OA on coccolithophores have shown diverse and sometimes contradictory results (for a review see [23]–[25]). Nevertheless, the majority of studies show calcification rates to generally decrease in response to OA while growth and carbon fixation rates had less clear trends. Factors potentially responsible for the discrepancies observed have been hypothesized to be related to different culturing conditions, such as temperature and light availability [23], [24], [26]–[30] and strain-specific responses [23], [31]. Thus, key uncertainties remain on the synergistic effects, and most importantly the understanding of underlying physiological mechanisms, of CO_2_ and other environmental factors on coccolithophore physiology. The main goal of our experiments is to understand whether increasing temperature can systematically induce changes in the carbonate chemistry sensitivity of growth, calcification and organic carbon fixation rates of *E. huxleyi* and *G. oceanica*.

## Materials and Methods

### Experimental set-up

Six experiments are presented in this study with generally similar design. Differences in experimental set-up were restricted to growth temperatures. Monospecific cultures of the coccolithophores *E. huxleyi* (strain PML B92/11 isolated from Bergen, Norway) and *G. oceanica* (strain RCC 1303 isolated from Arcachon Bay, France) were grown in dilute batch cultures [32] in a broad CO_2_ gradient (see carbonate chemistry) at three different temperature regimes. The temperature range for each strain was chosen according to its specific biogeographical distribution: 10, 15 and 20°C for *E. huxleyi* [33] and 15, 20 and 25°C for *G. oceanica* [34]. Temperatures will be referred from now on as: “low”, “intermediate” and “high”, respectively. Note that data points for the 15°C experiment with *E. huxleyi* were taken from the constant total alkalinity experiments on Bach et al. 2011 [28]. Cultures were incubated in a thermo constant (+/−0.05°C) climate chamber (RUMED Rubarth Apparate GmbH) at a light∶dark cycle of 16∶8 and photosynthetic active radiation (PAR) of 150 µmol m^−2^ s^−1^. Cells were pre-acclimated to experimental conditions for at least 7 generations which varied between 5–15 days depending on cell division rate. Initial cell densities varied between 50–600 cells ml^−1^ depending on treatment. The treatments with the highest cell densities were those at either extremes of the CO_2_ range since those had the lowest growth rates. Final cell densities in the main experiments did not exceed 30,000 cells ml^1^, minimizing dissolved inorganic carbon drawdown to less than 8% and preventing major changes in the carbonate chemistry speciation [32].

### Growth medium

All cultures were grown in artificial seawater (ASW) prepared as described in Kester et al. 1967 [35] but initially without the addition of sodium carbonate (see below carbonate chemistry). ASW was enriched with 64 µmol kg^−1^ nitrate, 4 µmol kg^−1^ phosphate, trace metals and vitamins (according to f/4 Guillard and Ryther 1962 [36]), 10 nmol kg^−1^ selenium, 2 ml kg^−1^ of 0.2 µm filtered North Sea water.

The nutrient enriched ASW solution (free of dissolved inorganic carbon, DIC, and total alkalinity, TA) was sterile filtered through a 0.2 µm nylon membrane (Whatman® Polycap ™ 75 AS) directly into acclimation (0.5 L Nalgene™) or experimental bottles (2 L Nalgene™) leaving only minimal headspace.

### Carbonate chemistry

Carbonate chemistry parameters, TA and DIC, were added to the culture medium by calculated additions of hydrochloric acid (certified HCl with a concentration of 3.571 mol L^−1^, Merck) and Na_2_CO_3_ (Merck, Suprapur quality and dried for 2 hours at 500°C). This resulted in a DIC gradient of ∼1300–2300 µmol kg^−1^ at constant TA, ∼2325±40 µmol kg^−1^. Samples for TA were taken at the beginning and end of the experiment, respectively. TA samples were filtered through GF/F filters, stored at 4°C and processed within 14 days. Otherwise samples were poisoned with HgCl_2_. TA samples were measured in a Metrohm Basic Titrino 794 titration device according to Dickson et al. 2003 [37].

**Figure 1:**
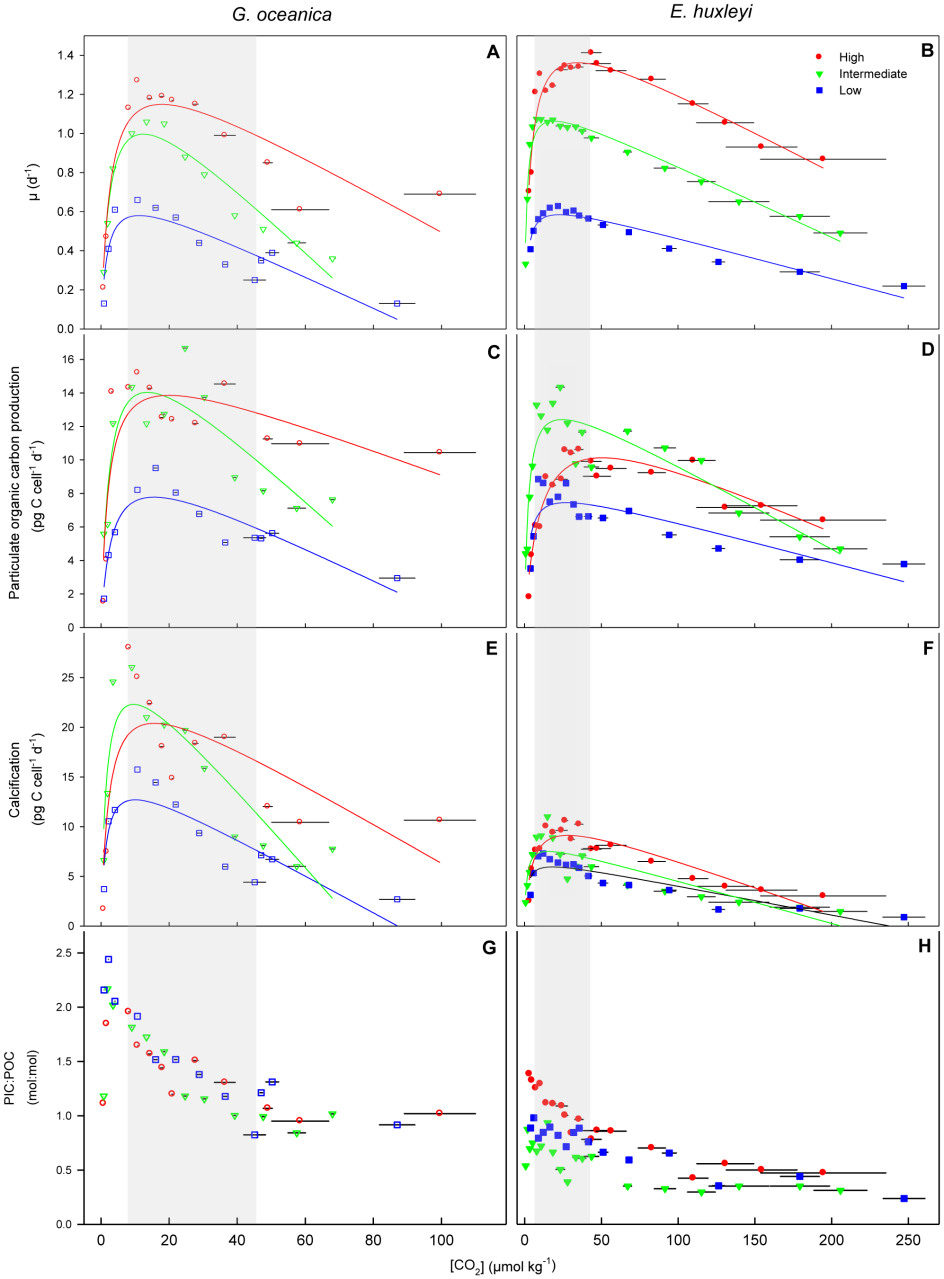
Physiological response of *G. oceanica* and *E. huxleyi* to increasing CO_2_ and temperature. Response of growth, POC production, calcification rates and PIC:POC to increasing CO_2_ and temperature of *G. oceanica* (left, open symbols) and *E. huxleyi* (right, closed symbols). Horizontal bars indicate change of CO_2_ from beginning to end of experiment. In some cases the changes were small and thus appear absent. Shaded areas represent OA relevant ranges (∼280–1000 µatm *p*CO_2_). Note that the investigated CO_2_ range (x-axis) is only half as broad for experiments with *G.oceanica* compared to the one of *E.huxleyi*.

Unfortunately, DIC samples were lost due to storing problems and had to be estimated from TA and acid additions as described in Bach et al. 2011 [28]. Briefly, initial DIC concentrations (DIC_initial_) were estimated as: 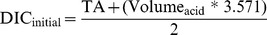
(1)where TA refers to the measured initial total alkalinity (µmol kg^−1^) and Volume_acid_ refers to the amount of acid added (µl kg^−1^) and 3.571 being the molarity of the acid (µmol µl^−1^). The accuracy of this method is explained in detail in Bach et al. 2011 [28] and shows that the maximum offset of ∼30 µmol kg^−1^ between DIC calculated with equation 1 and DIC calculated from measured TA and pH is small relative to the large DIC ranges (∼1300–2600 µmol kg^−1^). Final DIC concentrations were calculated by subtracting the measured total particulate carbon build-up from DIC_initial_ calculated with equation 1, assuming that the potential error from dissolved organic matter production is small compared to the broad ranges in DIC concentrations [28]. Carbonate chemistry parameters (pH, HCO_3_^−^, CO_3_^2−^, CO_2_) were calculated using the program CO2SYS (Lewis and Wallace 1998 [38]) from measured TA, estimated DIC, temperature, salinity and [PO_4_^3−^], and the dissociation constants determined by Roy et al. 1993. pH values are given on the free scale. Note that horizontal error bars on all figures denote for the change of CO_2_ concentrations during the course of the experiments and that biological response data is plotted against the average of initial and final [CO_2_]. The degree of change in carbonate chemistry conditions from beginning to end of the experiment depended on final cell densities. In some cases, final cell densities were not high and changes in carbonate chemistry were so small that they appear absent in Figure 1.

### Particulate organic and inorganic carbon

Sampling started two hours after the onset of the light period and lasted no longer than 2 hours. Samples for particulate carbon were filtered (200 mbar) onto GF/F filters (Whatmann, combusted at 500°C for 6 hours) and stored in glass petri- dishes (pre-combusted at 500°C for 6 hours) at -20°C until analysis. Filters for total particulate carbon (TPC) were dried overnight at 60°C while filters for particulate organic carbon (POC) were first placed in a desiccator above fuming (37%) HCl for 2 hours to remove all inorganic particulate carbon (PIC) and subsequently dried overnight at 60°C. Both TPC and POC samples were analyzed with a EuroEA analyzer according to Sharp 1974 [39] except for experiments with *E. huxleyi* at 15°C and *G. oceanica* at 25°C, for which TPC and POC were measured with an Elemental analyzer coupled to an isotope ratio mass spectrometer (EuroEA, Hekatech GmbH). Both methods use the same analysis but the latter is coupled to an isotope mass spectrometer which gives additional information on isotopic composition, if desired. PIC was calculated by subtracting POC from TPC.

### Growth

Cell densities were determined at the beginning and end of the experiment with a Beckman Z2 Coulter®Particle Count and Size Analyzer. Growth rates (μ) were calculated according to: 
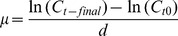
(2)where C_t-final_ represents cell densities at the end of the experiment, C_t0_ represents initial cell densities and d represents the number of days between t_0_ and t_final_. Calcification and POC production rates were obtained by multiplying growth rates with particulate inorganic or organic carbon cellular quotas, respectively.

### Statistics

A nonlinear regression model was used to analyze the data, with a higher number of treatment levels at the expense of replication. This approach is more informative for ecological modelling than the analysis of variance (ANOVA), nevertheless without the loss of statistical power[40]. The Michaelis-Menten kinetics can be used to describe the increase of growth and organic and inorganic carbon production with increasing CO_2_ concentrations levelling off at saturating conditions: 
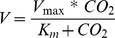
(3)where V is the growth or production rate at a certain CO_2_ concentration, V_max_ is the maximum growth or production rate and K_m_ is the CO_2_ concentration at which the rate is half-saturated. In our experiments growth and production rates decreased after reaching maximum values and thus a linear term was subtracted from equation 3 and the Michaelis-Menten equation modified as follow:
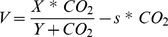
(4)where X and Y are random fit parameters and s the ‘sensitivity’ constant which describes the linear negative effect of increasing CO2 (decreasing pH)[28]. From the differentiated fit equation (explained in detail in [28]) we calculated optimum CO2 concentrations for growth, photosynthesis and calcification rates as well as maximum production rates (Vmax) and half saturation constants (Km). Coefficients of determination (R2), p-values, F-values and degrees of freedom for each fit are presented in Table 1 and 2. In the following sections optimum and maximum production rates refer to those calculated from the fit equation. Note that in some cases the highest measured values deviated from those determined using the fit equation. The first derivative of eq. 4 was calculated to detect temperature-dependent changes in calcification at specific [CO2] as follow:
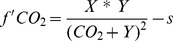
(5)a positive value of the first derivative (i.e. a positive slope of a tangent at certain [CO2]) indicates that there is a positive correlation between increasing CO2 and the investigated metabolic process while a negative value indicates the opposite. We calculated our values at [CO2] of 18 µmol kg−1, approximately 375–575 µatm pCO2, depending on temperature, and thus were able to compare the different temperature-dependent response at the same CO2 concentration.

**Table 1:** Parameters from fit equation (4) for *G. oceanica*.

G.oceanica			
Seasonal T_min_ = 7°C; seasonal T_max_ = 27°C [34]	Low	Intermediate	High
***CO_2_ optima (µmol kg^−1^)***	**15°C**	**20°C**	**25°C**
growth rate	11.3	12.4	17.8
POC production	15.6	13.6	20.0
calcification	10.1	9.6	15.6
***pCO_2_ optima (µatm)***			
growth rate	305	403	609
POC production	421	420	835
calcification	269	295	555
***V_max_***			
growth rate (d^−1^)	0.6	1.0	1.1
POC production (pg C cell^−1^d^−1^)	7.8	14.0	13.9
calcification (pg C cell^−1^d^−1^)	12.7	22.3	20.4
***K_m_ (µmol kg^−1^)***			
growth rate	1.1	1.4	1.7
POC production	1.8	1.5	1.6
calcification	1.0	1.0	1.5
***R^2^ (p-value)***			
growth rate	0.74 (0.0009)	0.92 (<0.0001)	0.83 (0.0001)
POC production	0.81 (0.0002)	0.70 (0.0018)	0.69 (0.0012)
calcification	0.69 (0.0021)	0.77 (0.0006)	0.58 (0.0081)
***F value (degrees of freedom)***			
growth rate	16.7 (11)	69.1 (11)	27.7 (11)
POC production	24.7 (11)	13.8 (11)	14.1 (12)
calcification	13.3 (11)	19.1 (11)	8.6 (11)
***sensitivity constant***			
growth rate	0.008	0.017	0.010
POC production	0.100	0.191	0.076
calcification	0.189	0.398	0.203

**Table 2:** Parameters from fit equation (4) for *E. huxleyi*.

E.huxleyi			
Seasonal T_min_ = 2°C; seasonal T_max_ = 16°C [33]	Low	Intermediate	High
***CO_2_ optima (µmol kg^−1^)***	**10°C**	**15°C**	**20°C**
growth rate	22.6	19.3	34.9
POC production	27.3	24.5	50.6
calcification	18.1	15.4	28.4
***pCO_2_ optima (µatm)***			
growth rate	519	622	1295
POC production	612	656	1535
calcification	412	414	875
***V_max_***			
growth rate (d^−1^)	0.6	1.1	1.4
POC production (pg C cell^−1^d^−1^)	7.5	12.4	10.1
calcification (pg C cell^−1^d^−1^)	5.9	7.5	9.1
***K_m_ (µmol kg^−1^)***			
growth rate	1.4	1.0	2.6
POC production	1.8	1.8	5.9
calcification	1.2	1.0	3.0
***R^2^ (p-value)***			
growth rate	0.90 (<0.0001)	0.95 (<0.0001)	0.90 (<0.0001)
POC production	0.58 (0.0013)	0.81 (<0.0001)	0.87 (<0.0001)
calcification	0.74 (<0.0001)	0.69 (<0.0001)	0.81 (<0.0001)
***F value (degrees of freedom)***			
growth rate	69.3 (15)	166.9 (18)	81.7 (17)
POC production	11.5 (15)	39.8 (18)	60.3 (17)
calcification	22.2 (15)	20.7 (18)	38.2 (17)
***sensitivity constant***			
growth rate	0.002	0.004	0.004
POC production	0.024	0.051	0.042
calcification	0.030	0.043	0.055

## Results

The coccolithophores *E. huxleyi* and *G. oceanica* were incubated in a broad CO_2_ gradient and three different temperatures. All investigated metabolic processes in both species displayed an optimum-curve response pattern along the CO_2_ gradient. However, temperature clearly modified the shape of this response (Figure 1).

Optimum CO_2_ concentrations, V_max_ and K_m_ for growth, photosynthetic carbon fixation and calcification rates were assessed with fit equation (4). Resulting V_max_, K_m_ and *p*CO_2_/CO_2_ optima as well as statistical results (coefficients of determination (R^2^), p-values, F values and degrees of freedom) are presented in Table 1 and 2. The underlying carbonate chemistry is presented in the supplement Tables S1–S2 and shows the average values from initial and final calculated CO_2_, *p*CO_2_, DIC, pH_free_ and ΩCa. Physiological responses (i.e. growth, POC production, calcification rates and PIC∶POC ratios) are also given in supporting information Table S1–S2.

### Growth rates (μ)

Increasing temperature stimulated growth rates of *E. huxleyi* and *G. oceanica* (Figure 1A–B). Approximately a doubling in maximum growth rates was observed in both species between the lowest and highest temperature tested (Figure 1 A–B, Table 1–2). The CO_2_ half-saturation for growth slightly but constantly increased in *G. oceanica* throughout the investigated temperature range (Table 1). In *E. huxleyi*, CO_2_ half-saturation slightly decreased from low to intermediate temperatures but increased strongly from intermediate to high temperature (Table 2). Optimum growth for *G. oceanica* was observed at [CO_2_] of ∼11–13 µmol kg^−1^ (∼300–400 µatm *p*CO_2_) at low and intermediate temperature and ∼18 µmol kg^−1^ (∼600 µatm *p*CO_2_) at high temperature (Table 1). Optima for *E. huxleyi* was observed at [CO_2_] of ∼19–23 µmol kg^−1^ (∼520–620 µatm *p*CO_2_) at low and intermediate temperature and ∼35 µmol kg^−1^ (∼1300 µatm *p*CO_2_) at high temperature (Table 2).

### POC production

POC production was stimulated from low to intermediate temperatures in both species (Figure 1C–D). The highest temperature did not enhance POC production in *G. oceanica* (Figure 1C) and reduced the rate in *E. huxleyi* (Figure 1D). CO_2_ half-saturation showed no clear trend in *G. oceanica* throughout the entire temperature range (Table 1) while the half-saturation remained unaffected *in E. huxleyi* from low to intermediate temperature but strongly increased at high temperature (Table 2). Optimum CO_2_ concentrations for POC production rates in *G. oceanica* were observed at ∼13–16 µmol kg^−1^ (∼420 µatm *p*CO_2_) at low and intermediate temperature and ∼20 µmol kg^−1^ (∼835 µatm *p*CO_2_) at high temperature (Table 1). The CO_2_ optima for *E. huxleyi* were found at ∼24–28 µmol kg^−1^ (∼600–650 µatm *p*CO_2_) at low and intermediate temperature and 50 µmol kg^−1^ (∼1535 µatm *p*CO_2_) at high temperature (Table 2).

### Calcification rates

Maximum calcification rates increased in *G. oceanica* from low to intermediate temperature but did not accelerate any further at high temperature levels (Figure 1E, Table 1). In *E. huxleyi*, maximum calcification rates increased steadily over the investigated temperature range (Figure 1F). The CO_2_ half-saturation showed in both species very little change from low to intermediate temperatures but increased substantially from intermediate to high temperature (Table 1–2). Optimum calcification rates for *G. oceanica* were observed at [CO_2_] of ∼10 µmol kg^−1^ (∼260–300 µatm *p*CO_2_) at low and intermediate temperature and at ∼16 µmol kg^−1^ (∼550 µatm *p*CO_2_) at the highest temperature (Table 1). For *E. huxleyi* optimum calcification rates were observed at [CO_2_] of ∼15–18 µmol kg^−1^ (∼400 µatm *p*CO_2_) at low and intermediate temperature and at ∼30 µmol kg^−1^ (∼875 µatm *p*CO_2_) at the highest temperature (Table 2).

Increasing temperature did not modulate the effect of increasing CO_2_ on the PIC∶POC ratio (Figure 1G–H). The PIC∶POC ratio was temperature-independent in *G. oceanica* and linearly decreased with increasing CO_2_ (Figure 1G). *G. oceanica* had a large fraction of the assimilated particulate carbon as inorganic carbon (calcite) which was then reflected in the higher PIC∶POC compared to *E. huxleyi* (Figure 1G–H). Although no clear trends could be observed for *E. huxleyi*, the highest PIC∶POC were found at the highest temperature while lowest ratios were observed at intermediate temperature and linearly decreased with a possible stabilization at [CO_2_] levels >100 µmol kg^−1^ (Figure 1H).

## Discussion

Ocean acidification and warming are two ongoing processes potentially affecting marine phytoplankton - in particular calcifying species [5]. Here, we investigated how temperature modulates the sensitivity of key metabolic processes (i.e. growth, photosynthesis and calcification) to increasing CO_2_ in the two most abundant coccolithophores *E. huxleyi* and *G. oceanica*. Our experiments confirm that the optimum-curve response of growth, photosynthesis and calcification obtained for *E. huxleyi* over a broad CO_2_ gradient [28] also holds under different temperature conditions. Likewise, this response pattern was also displayed by *G. oceanica* at all investigated temperatures.

Despite this similarity in the overall response pattern, the position of optima varied between temperature treatments. In most cases, optimum [CO_2_] where maximum rates were achieved was shifted towards higher CO_2_ concentrations with increasing temperature. This underlines the importance of temperature not only in determining absolute growth and production rates but also in systematically modulating sensitivities of these metabolic processes to changing seawater carbonate chemistry speciation.

Additionally, we observed species-specific sensitivities to increasing CO_2_ of *E. huxleyi* and *G. oceanica*, as previously pointed out by Riebesell et al. 2000 [22] and Zondervan et al. 2001 [41]. Optimum CO_2_ levels for calcification rates were remarkably lower than those for organic carbon fixation rates in both species (Tables 1–2). However, *G. oceanica* had a narrower CO_2_ tolerance range (compare x-axis in Figure 1) and thus the sensitivity constants obtained from the fit equation (4) were about four times higher than in *E. huxleyi* at similar overall rates (compare “sensitivity constant” Tables 1 and 2). Interestingly, sensitivity constants for calcification were about twice as high as those for organic carbon fixation, suggesting that the former process is more susceptible to changes in carbonate chemistry in the high CO_2_ range (Table 1). Potentially, *G. oceanica* is more representative of larger coccolithophore species with higher calcification than carbon fixation rates such as *C. leptoporus and C. braarudii* [42], [43]. For instance, *C. braarudii* stopped calcifying at levels higher than ∼80 µmol kg^−1^ CO_2_ (approx. 2380 µatm *p*CO_2_ [43]) while *C. leptoporus* had an optimum curve response in an even narrower range of ∼4–35 µmol kg^−1^ CO_2_ (approx. 180–1000 µatm *p*CO_2_ [42]) suggesting that higher CO_2_ concentrations (most likely concomitantly lower pH) would be more detrimental to highly calcifying strains compared to those with PIC∶POC ratios closer to 1 (i.e. *E. huxleyi*).

### Both coccolithophores show highest metabolic activity at temperatures significantly higher than experienced at isolation site

Stimulating effects of increasing temperature have been widely observed in phytoplankton through accelerating metabolic activities [44]–[46]. In accordance with this, we observed a pronounced acceleration of growth in both species within the investigated temperature range (Figure 1A–B). Maximum growth rates doubled for each 10°C increase, closely matching values reported in the literature for these two species between 6 and 25°C [13], [31], [47]–[49]. Interestingly, each species had highest growth rates at temperatures 5–10°C higher than the maxima observed at their isolation sites when they are the most abundant (Table 1–2 [33], [34]). Conte et al. 1998 [47] also observed that an *E. huxleyi* strain had the highest growth rates at temperatures 10°C above those occurring at the strain's natural environment. This could have several implications. Either their biogeographic distribution is relatively broad and both coccolithophore strains tested here occur in large areas which include locations with significantly higher maximum seasonal temperatures or both strains have physiological flexibility which allows them to exploit high temperature anomalies. Both of which, based on the assumption that the strain has optimized its temperature response to its specific environment. The biogeographic distribution and local temperature adaptation of coccolithophores has not been studied in great detail but recent evidence points towards a certain degree of isolation between coccolithophore populations [50], [51]. However, there appears the be a strong link between the local environment and the thermal tolerance of marine phytoplankton in the global ocean. Thomas et al. 2012 [52] showed that phytoplankton from polar and temperate regions had optimum growth at temperatures above the annual mean of their region and wider thermal tolerance curves while tropical phytoplankton had their optimum growth below (or at) their annual mean with narrower thermal tolerance curves. They suggested that phytoplankton in the tropics might have a greater risk to future warming scenarios due to their narrow thermal tolerance sensitivities. Nevertheless, for temperate and polar species, being able to exploit high temperature anomalies may be an ecological advantage, in particular for *E. huxleyi* which often blooms during early summer in highly stratified shallow mixed layers [53]. This could have been the case when a large *E. huxleyi* bloom occurred in the Bering Sea in 1997, a year characterized with a particularly shallow mixed layer depth, exceptionally high water temperatures (∼4°C above mean), and high irradiances [54]. Although the occurrence of such blooms was not observed regularly in this oceanic region, there was some indication that *E. huxleyi* progressively entered the region and benefited in this particular year from such climatic anomalies [55].

### Rising temperature affects organic carbon fixation and calcification differently

Maximum growth rates increased consistently with increasing temperature in both species suggesting that overall metabolism is generally accelerated within these temperature ranges (Figure 1 A–B; Table 1–2). Nevertheless, the temperature sensitivity of both species differed in terms of the maximum organic and inorganic carbon fixation rates. In *G. oceanica*, maximum organic carbon fixation increased strongly from low to intermediate temperature but showed little change thereafter (Figure 1C; Table 1). Maximum calcification rates showed a similar trend from low to intermediate temperatures but even slightly decreased towards the highest investigated temperature (Figure 1E; Table 1). In *E. huxleyi*, maximum organic carbon fixation nearly doubled from low to intermediate temperature but decreased thereafter, while maximum calcification rates increased linearly over the entire temperature range (Figure 1D, F; Table 2).

The importance of temperature for modulating resource allocation in marine phytoplankton was recently pointed out by Toseland et al. 2013 [56]. Results from their pair-wise gene ontology analysis, as well as their model for optimal resource allocation, indicated that at low temperatures (i.e. temperate and polar regions) cells would preferentially allocate resources into biosynthesis rather than photosynthesis leading to higher allocation of P into rich rRNA and a subsequent decrease of the N∶P stoichiometry. Antagonistically, at warmer temperatures resource allocation would preferentially go into photosynthesis and thus higher N∶P ratios.

In our experiments, a re-distribution of inorganic carbon among requiring pathways (i.e. photosynthesis and/or calcification) could have occurred. This becomes particularly apparent in *E. huxleyi* at the high temperature treatment (i.e. 20°C) where maximum POC fixation rate clearly has a lower temperature optimum than calcification rate (compare Figure 1D and F; Table 2). Whereas total particulate carbon was similar between the high and intermediate temperature treatments, a high PIC∶POC suggested that inorganic carbon was incorporated towards calcification rather than photosynthesis at the high temperature treatment (Figure 1H). Indeed, it was recently postulated that changes in carbonate chemistry affect the redox state inside *E. huxleyi* cells which subsequently causes a reorganization of carbon flux networks within and across cellular compartments (e.g. cytosol, chloroplast and calcification vesicle) [57]. Increasing *p*CO_2_, for example, diverted inorganic carbon away from calcification into pathways involved in the production of organic carbon [30], [57].

In this respect it is worth noting that cellular PIC∶POC in coccolithophores is a measure for carbon allocation to photosynthesis and calcification and cannot be used to infer potential feedbacks to atmospheric carbon dioxide levels. The latter is ultimately affected by the PIC∶POC of export production and while about half of pelagic PIC stems from coccolithophores most of the POC is contributed by other phytoplankton groups such as diatoms. Therefore, it is the changes in the absolute amount of PIC produced by coccolithophores and not in cellular PIC∶POC which need to be considered for global biogeochemical element cycling.

### Changing temperature shifts the optimum CO_2_ concentration for growth, photosynthesis and calcification

**Figure 2:**
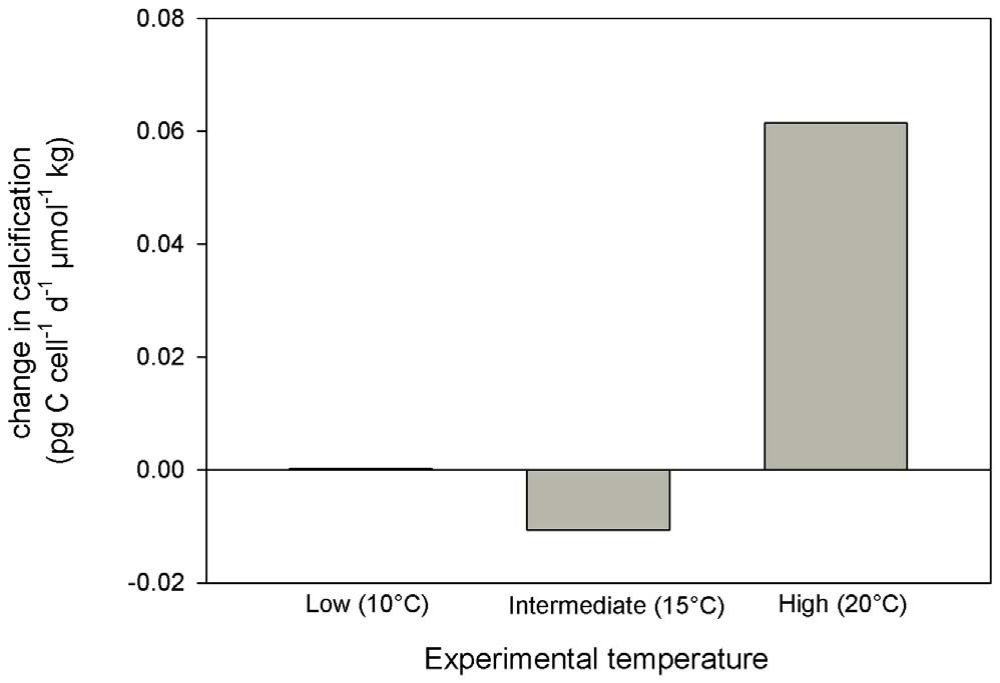
Calcification rate of *E. huxleyi* in response to elevated CO_2_ at different temperatures. Depending on the growth temperature the rate of calcification can decrease strongly or moderately or even increase with rising CO_2_ levels. The “low”, “intermediate” and “high” refers to experimental temperature of 10, 15 and 20°C, respectively. The slope of a tangent at [CO_2_] of 18 µmol kg^−1^ in the 10°C treatment of *E. huxleyi* is almost 0 which means that the optimum curve has reached a plateau in the OA relevant CO_2_ range. At 20°C there is a positive slope which means that cells have not yet reached the optimum CO_2_ for calcification at 18 µmol kg^−1^ in this temperature.

**Figure 3:**
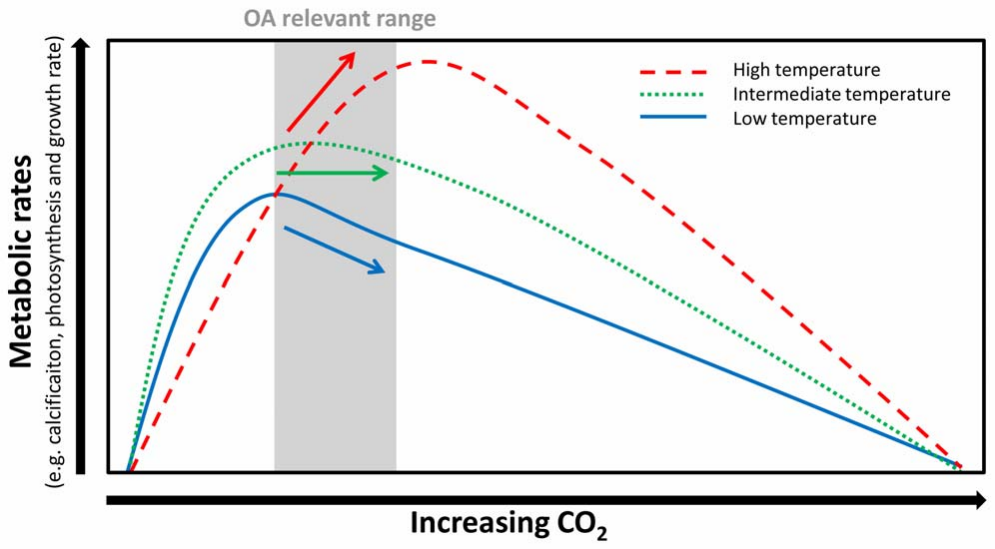
Conceptual graph depicting the modulating effect of temperature on the CO_2_/pH sensitivity of key metabolic rates in coccolithophores. The arrows emphasize the trends of key metabolic processes (i.e. calcification, photosynthesis and growth) vs. CO_2_ relationship in the range relevant to future ocean acidification.

CO_2_ optima for growth, organic carbon fixation and calcification rates shifted towards higher CO_2_ concentrations from intermediate to high temperatures in both coccolithophores examined in this study (Table 1–2). The temperature-induced optimum shift in *E. huxleyi* was even strong enough to reverse the response of calcification rates within the ocean acidification relevant range (i.e. ∼7–45 µmol kg^−1^ CO_2_, approx. 280–1000 µatm *p*CO_2_) from negative at low and intermediate temperatures to positive at high temperature (Figure 2). Hence, a coccolithophore strain can respond to ocean acidification, with both, increasing and decreasing calcification rate, depending on the temperature it was cultured at. This has major implications when trying to compare the responses of different coccolithophore strains cultured at different experimental settings to increasing seawater CO_2_. To account for the modulating effect of temperature on the CO_2_/pH sensitivity, a direct comparison of species/strain specific responses should occur in the same range of their temperature optimum curves. Investigating the effect of ocean acidification on metabolic processes in coccolithophores therefore requires a sound understanding of the influence of experimental culture conditions (in this study temperature conditions but the same probably also applies to other parameters such as light [30]). If this understanding is missing, then interpretation of the response of coccolithophores to future ocean *p*CO_2_ from laboratory incubations can be misleading because all responses (increase, plateau, decrease) within the OA-relevant *p*CO_2_ range can be attained when growing the investigated cells at certain culture conditions (Figure 3).

The reason for the observed CO_2_ optimum shift for growth, organic carbon fixation and calcification at the highest temperature is presently unclear. Possibly the strongly accelerated metabolism at high temperatures, and concomitantly higher demand of inorganic carbon, benefits more from the increased CO_2_ substrate availability than it is negatively affected by the increase in proton concentrations (low pH). Or in other words, the balance between a fertilizing effect of additional inorganic carbon and a metabolic repression by high proton concentration observed in previous studies [28], [58] is apparently modulated by increasing temperature.

In our study, increasing temperature partly compensates the negative effect of increasing proton concentrations on calcification rates to some extent. However, Feng et al. 2008[59] and De Bodt et al. 2010 [60] found the opposite – i.e. a negative feedback of increasing temperature on calcification rates under high CO_2_ conditions at very different temperature regimes (13 and 18°C and 20 and 24°C, respectively). This apparent contradiction can potentially be resolved when considering the diverse temperature sensitivities among strains [47], [49]. In case increasing temperature stimulates the investigated strain (as in our study) a partial compensation of the negative influence of high proton concentrations seems to be likely. However, as soon as increasing temperature becomes a stress factor, excess proton concentrations may exacerbate the temperature effect. In the latter case, rising temperature and proton concentration could synergistically reduce metabolic rates in coccolithophores.

## Supporting Information

Table S1 Carbonate chemistry and physiological parameters for *G. oceanica*.
(PDF)

Table S2 Carbonate chemistry and physiological parameters for *E. huxleyi*.
(PDF)
