# Supplementary material for: Temperature Modulates Coccolithophorid Sensitivity of Growth, Photosynthesis and Calcification to Increasing Seawater pCO2
Source: PLoS One. 2014 Feb 5;9(2):e88308. doi: 10.1371/journal.pone.0088308 (PMC3914986; doi:10.1371/journal.pone.0088308)
Supplement: Table S1 — Carbonate chemistry and physiological parameters for G. oceanica. (PDF) [file pone.0088308.s001.pdf]

Table S1. Carbonate chemistry and physiological parameters for *G. oceanica*

Temp. (°C)	CO_2 (µmol kg^{-1})	pCO_{2ave} (µatm)	TA_{ave} (µmol kg^{-1})	DIC_{ave} (µmol kg^{-1})	pH_{free}	Ω Ca	µ (d^{-1})	POC production (pg C cell^{-1} d^{-1})	Calcification (pg C cell^{-1} d^{-1})	PIC:POC
**15**	0.9	24	2330.7	1456.0	8.93	14.08	0.13	1.72	3.71	2.16
	2.2	59	2301.8	1637.2	8.68	10.76	0.41	4.32	10.53	2.44
	4.1	110	2275.8	1765.5	8.49	8.29	0.61	5.68	11.68	2.05
	10.7	286	2313.6	2010.5	8.17	5.08	0.66	8.22	15.75	1.92
	16.0	430	2284.9	2065.8	8.02	3.79	0.62	9.52	14.45	1.52
	21.9	588	2295.4	2128.6	7.91	3.03	0.57	8.05	12.23	1.52
	28.8	773	2286.8	2163.7	7.80	2.43	0.44	6.78	9.35	1.38
	36.5	977	2297.0	2206.5	7.71	2.02	0.33	5.07	5.97	1.18
	45.1	1209	2289.7	2228.6	7.62	1.69	0.25	5.35	4.41	0.82
	47.1	1261	2342.8	2283.8	7.61	1.69	0.35	5.33	7.11	1.21
	50.3	1348	2365.9	2314.5	7.59	1.63	0.39	5.63	6.71	1.31
	87.0	2331	2281.2	2308.0	7.35	0.93	0.13	2.94	2.70	0.92
**20**	0.8	25	2339.8	1420.8	8.90	14.83	0.29	5.58	6.60	1.18
	1.9	59	2295.6	1580.7	8.66	11.64	0.54	6.16	13.37	2.17
	3.5	108	2287.9	1718.6	8.48	9.35	0.82	12.18	24.58	2.02
	9.0	280	2290.6	1941.7	8.17	5.88	1.00	14.34	26.03	1.81
	13.3	413	2280.4	2014.3	8.04	4.58	1.06	12.17	21.00	1.73
	18.5	574	2268.5	2065.5	7.91	3.63	1.05	12.74	20.26	1.59
	24.7	764	2278.2	2122.5	7.81	2.95	0.88	16.68	19.71	1.18
	30.3	940	2308.6	2181.9	7.73	2.57	0.79	13.74	15.88	1.16
	39.3	1216	2296.1	2208.8	7.63	2.06	0.58	8.95	8.99	1.00
	47.7	1476	2307.1	2246.1	7.55	1.77	0.51	8.16	8.09	0.99
	57.5	1781	2302.7	2267.8	7.48	1.50	0.44	7.13	6.00	0.84
	68.0	2104	2313.7	2301.1	7.41	1.30	0.36	7.63	7.76	1.02
**25**	0.7	26	2261.9	1335.1	8.85	14.72	0.21	1.54	1.71	0.10
	1.7	58	2385.3	1583.1	8.65	13.25	0.47	4.05	7.47	0.31
	8.1	288	2267.2	1883.0	8.15	6.50	1.13	14.32	28.02	2.20
	10.7	377	2212.8	1898.8	8.05	5.35	1.27	15.21	25.05	2.84
	14.4	509	2204.2	1952.0	7.94	4.39	1.18	14.28	22.41	3.25
	18.0	635	2215.1	2001.8	7.86	3.81	1.19	12.53	18.06	3.29
	20.9	740	2242.5	2052.3	7.81	3.49	1.17	12.41	14.86	3.55
	27.7	980	2264.0	2117.8	7.71	2.88	1.15	12.18	18.37	4.23
	36.4	1284	2241.5	2138.6	7.60	2.29	0.99	14.53	18.99	6.35
	49.0	1731	2247.7	2187.6	7.48	1.79	0.85	11.25	12.02	6.29
	58.5	2067	2227.0	2192.6	7.41	1.52	0.61	10.96	10.43	7.20
	99.5	3517	2017.7	2067.3	7.15	0.78	0.69	10.44	10.65	13.43
