# Supplementary material for: Temperature Modulates Coccolithophorid Sensitivity of Growth, Photosynthesis and Calcification to Increasing Seawater pCO2
Source: PLoS One. 2014 Feb 5;9(2):e88308. doi: 10.1371/journal.pone.0088308 (PMC3914986; doi:10.1371/journal.pone.0088308)
Supplement: Table S2 — Carbonate chemistry and physiological parameters for E. huxleyi. (PDF) [file pone.0088308.s002.pdf]

Table S2. Carbonate chemistry and physiological parameters for *E. huxleyi*

**Temp.**	**CO_2**	***p* CO_{2ave}**	**TA_{ave}**	**DIC_{ave}**	**pH_{free}**	**Ω Ca**	**μ**	**POC production**	**Calcification**	**PIC:POC**
**(°C)**	**(μmol kg^{-1})**	**(μatm)**	**(μmol kg^{-1})**	**(μmol kg^{-1})**			**(d^{-1})**	**(pg C $cell^{-1}$ d^{-1})**	**(pg C $cell^{-1}$ d^{-1})**	
**10**	3.9	89	2327.5	1809.7	8.57	8.4	0.42	3.52	3.12	0.89
	5.9	136	2345.6	1923.8	8.44	6.9	0.52	5.44	5.33	0.98
	8.9	204	2341.1	2011.4	8.30	5.4	0.58	8.86	7.02	0.79
	12.1	277	2345.3	2077.6	8.19	4.5	0.61	8.63	7.29	0.85
	16.4	375	2334.6	2125.2	8.08	3.6	0.64	7.51	6.72	0.90
	21.7	497	2319.0	2159.0	7.97	2.9	0.65	7.80	6.38	0.82
	27.0	619	2311.5	2185.7	7.88	2.4	0.62	8.62	6.15	0.71
	31.9	730	2322.9	2220.1	7.82	2.1	0.63	7.34	6.21	0.85
	35.6	813	2326.8	2238.4	7.78	2.0	0.60	6.62	5.86	0.89
	41.5	950	2325.3	2258.0	7.72	1.7	0.58	6.63	5.03	0.76
	51.1	1170	2330.7	2290.1	7.63	1.4	0.55	6.54	4.33	0.66
	68.0	1556	2334.2	2330.6	7.51	1.1	0.51	6.96	4.12	0.59
	94.3	2156	2341.0	2381.1	7.38	0.8	0.43	5.53	3.62	0.66
	126.3	2890	2341.9	2426.5	7.26	0.6	0.35	4.72	1.67	0.35
	179.3	4103	2348.5	2497.1	7.11	0.5	0.30	4.04	1.78	0.44
	247.2	5656	2348.4	2572.6	6.97	0.3	0.23	3.78	0.89	0.24
**15**	0.7	20	2334.10	1420.8	8.98	14.7	0.34	4.41	2.37	0.54
	1.9	50	2324.41	1612.0	8.73	11.6	0.69	4.67	4.09	0.87
	3.4	90	2318.5	1748.6	8.56	9.3	0.98	7.78	5.40	0.69
	5.3	141	2315.3	1854.3	8.42	7.6	1.07	9.63	7.20	0.75
	7.8	210	2309.8	1941.6	8.28	6.1	1.11	13.27	8.94	0.67
	10.8	289	2292.5	1995.9	8.17	5.0	1.11	12.64	9.09	0.72
	14.9	400	2297.6	2063.8	8.05	4.0	1.09	11.79	11.00	0.93
	18.4	494	2302.0	2104.8	7.98	3.5	1.11	13.39	8.90	0.66
	23.3	624	2288.8	2131.4	7.89	2.9	1.07	14.35	7.23	0.50
	27.9	747	2304.8	2175.0	7.82	2.5	1.07	12.19	4.75	0.39
	33.4	894	2294.8	2192.3	7.74	2.2	1.07	9.78	6.03	0.62
	37.6	1007	2314.3	2226.9	7.70	2.0	1.05	11.66	7.07	0.61
	43.6	1169	2302.5	2236.1	7.64	1.8	1.01	9.59	5.98	0.62
	67.1	1797	2291.5	2283.2	7.46	1.2	0.94	11.73	4.11	0.35
	91.4	2450	2294.1	2327.9	7.33	0.9	0.85	10.73	3.51	0.33
	115.2	3086	2306.9	2374.7	7.24	0.7	0.78	9.97	2.95	0.30
	139.7	3742	2319.4	2418.2	7.16	0.6	0.67	6.84	2.40	0.35
	179.3	4804	2312.6	2459.5	7.05	0.5	0.60	5.41	1.90	0.35
	205.8	5513	2336.8	2513.6	7.00	0.4	0.51	4.69	1.46	0.31
**20**	3.0	94	2343.0	1708.0	8.53	10.1	0.73	1.82	2.52	1.38
	4.7	146	2330.3	1813.9	8.39	8.4	0.83	4.32	5.72	1.32
	7.3	225	2324.6	1906.0	8.25	6.7	1.25	6.08	7.62	1.25
	10.2	314	2323.5	1975.4	8.14	5.6	1.35	6.00	7.76	1.29
	13.9	431	2317.9	2038.8	8.02	4.5	1.26	8.99	10.03	1.12
	18.5	572	2321.2	2092.3	7.92	3.8	1.29	8.48	9.40	1.11
	24.1	746	2318.9	2126.7	7.82	3.1	1.37	8.84	9.62	1.09
	26.4	816	2341.1	2175.0	7.79	2.9	1.39	10.59	10.61	1.00
	30.5	945	2336.3	2196.7	7.73	2.6	1.38	10.40	8.73	0.84
	35.4	1097	2327.5	2211.1	7.67	2.3	1.38	10.62	10.24	0.96
	43.5	1347	2253.2	2181.4	7.58	1.9	1.46	9.91	7.74	0.78
	47.2	1461	2328.6	2237.2	7.56	1.8	1.40	9.03	7.78	0.86
	56.4	1746	2331.5	2272.3	7.49	1.6	1.37	9.49	8.13	0.86
	82.8	2565	2247.7	2264.4	7.32	1.0	1.32	9.25	6.49	0.70
	109.8	3400	2281.9	2338.9	7.21	0.8	1.19	9.97	4.77	0.42
	130.8	4049	2258.0	2343.5	7.13	0.7	1.09	7.16	3.99	0.56
	154.4	4781	2320.0	2417.8	7.07	0.6	0.96	7.26	3.62	0.50
	194.6	6025	2318.7	2459.2	6.97	0.5	0.90	6.40	3.03	0.47
